# Developmental Enhancement of Adenylate Kinase-AMPK Metabolic Signaling Axis Supports Stem Cell Cardiac Differentiation

**DOI:** 10.1371/journal.pone.0019300

**Published:** 2011-04-27

**Authors:** Petras P. Dzeja, Susan Chung, Randolph S. Faustino, Atta Behfar, Andre Terzic

**Affiliations:** Marriott Heart Disease Research Program, Division of Cardiovascular Diseases, Departments of Medicine, Molecular Pharmacology and Experimental Therapeutics, and Medical Genetics, Mayo Clinic, Rochester, Minnesota, United States of America; City of Hope National Medical Center and Beckman Research Institute, United States of America

## Abstract

**Background:**

Energetic and metabolic circuits that orchestrate cell differentiation are largely unknown. Adenylate kinase (AK) and associated AMP-activated protein kinase (AMPK) constitute a major metabolic signaling axis, yet the role of this system in guiding differentiation and lineage specification remains undefined.

**Methods and Results:**

Cardiac stem cell differentiation is the earliest event in organogenesis, and a suitable model of developmental bioenergetics. Molecular profiling of embryonic stem cells during cardiogenesis revealed here a distinct expression pattern of adenylate kinase and AMPK genes that encode the AK-AMP-AMPK metabolic surveillance axis. Cardiac differentiation upregulated cytosolic AK1 isoform, doubled AMP-generating adenylate kinase activity, and increased AMP/ATP ratio. At cell cycle initiation, AK1 translocated into the nucleus and associated with centromeres during energy-consuming metaphase. Concomitantly, the cardiac AMP-signal receptor AMPKα2 was upregulated and redistributed to the nuclear compartment as signaling-competent phosphorylated p-AMPKα(Thr172). The cardiogenic growth factor TGF-β promoted AK1 expression, while knockdown of AK1, AK2 and AK5 activities with siRNA or suppression by hyperglycemia disrupted cardiogenesis compromising mitochondrial and myofibrillar network formation and contractile performance. Induction of creatine kinase, the alternate phosphotransfer pathway, compensated for adenylate kinase-dependent energetic deficits.

**Conclusions:**

Developmental deployment and upregulation of the adenylate kinase/AMPK tandem provides a nucleocytosolic energetic and metabolic signaling vector integral to execution of stem cell cardiac differentiation. Targeted redistribution of the adenylate kinase-AMPK circuit associated with cell cycle and asymmetric cell division uncovers a regulator for cardiogenesis and heart tissue regeneration.

## Introduction

Energetic and metabolic signaling circuits are critical for organ function from embryonic development, and throughout lifespan [1], [2], [3], [4], [5], [6], [7], [8]. Cardiac specification and differentiation of stem cells is the earliest event in organogenesis requiring coordinated organization of the metabolic infrastructure to meet energy demands of the newly formed heart tissue [9], [10], [11], [12], [13], [14], [15]. Cardiogenic differentiation mandates robust metabolic signaling and information exchange between mitochondria and cytosolic/nuclear compartments to ensure developmental programming and an energetic continuum that sustains the function of nascent cardiomyocytes [7], [16], [17], [18].

Underlying the transition from low-energy requiring pluripotency into a cardiac phenotype is a switch in energy metabolism, from anaerobic glycolysis to more efficient mitochondrial oxidative phosphorylation [9], [19], [20]. Glycolytic and creatine kinase network formation provides energetic connectivity between expanding mitochondrial clusters and ATP-utilization cellular sites [9], [17], [21]. Despite advances in decoding the dynamics of major ATP production and distribution processes during lineage specification, metabolic signaling circuits responsible for integration of energetic events with cardiogenic programming remain largely unknown.

Adenylate kinase phosphorelays are recognized facilitators of metabolic signaling, optimizing intracellular energetic communication and local ATP supply [7], [22], [23]. The unique property of adenylate kinase catalysis to transfer both β- and γ-phosphoryls doubles the energetic potential of the ATP molecule, and provides a thermodynamically efficient mechanism for high-energy phosphoryl transport from mitochondria to myofibrils and the cell nucleus [7], [16], [24], [25], [26], [27]. Recent studies indicate that mitochondrial adenylate kinase (AK2) is required for unfolded protein response [28] and that AK2 deficiency compromises embryonic development and hematopoiesis by interfering with mitochondrial ATP/ADP exchange [29], [30], [31]. In this regard, the stress-responsive adenylate kinase isoform network, coupled with AMP signaling through AMP-activated kinase (AMPK), provides high-fidelity surveillance of energy metabolism to sustain the balance of energy supply and demand [7], [22], [32]. The metabolic sensor AMPK appears essential for embryonic development, maintaining cell polarity and cell cycle progression [18], [33], [34], [35], [36], and the upstream kinase LKB1 is critical for cardiac development, and in hematopoietic stem cell maintenance and cell division [37], [38], [39], [40]. However, the contribution of the adenylate kinase/AMPK tandem in stem cell cardiac differentiation has not been determined.

Here, we uncovered a developmental deployment and upregulation of the integrated adenylate kinase and AMP-AMPK signaling system underlying the execution of cardiogenic programming during embryonic stem cell differentiation. Nuclear translocation of adenylate kinase and p-AMPK supported energy-dependent cell division, and facilitated asymmetric differentiation leading to cardiac specification. Targeted knockdown of the adenylate kinase-dependent energetic and AMP signaling cascade disrupted maturation of mitochondrial networks and myofibrillogenesis, precluding formation and function of organized cardiac beating structures.

## Results and Discussion

### Restructuring of the adenylate kinase isoform network in stem cell cardiogenesis

**Figure 1:**
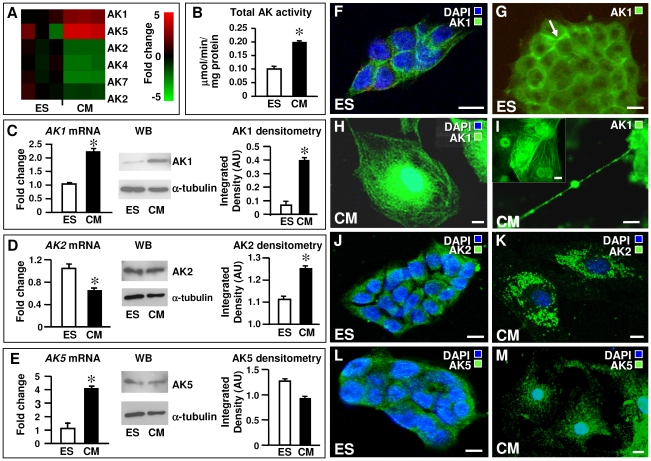
Cardiac differentiation is associated with enhancement and restructuring of the adenylate kinase isoform network. **A** - Microarray analyses of total mRNA in embryonic stem (ES) cells and derived cardiomyocytes (CM). Adenylate kinase isoform genes are hierarchically clustered as mRNA copy numbers of CM *versus* ES transcripts with extent of fold change color-coded (n = 3 in each group). **B** – ES cells have lower total adenylate kinase activity than derived CM. **C** – CM have more AK1 transcripts and protein than ES cells. **D and E** - CM transcribe AK2 less and AK5 more frequently than ES cells but protein levels of both isoforms are approximately equal. **F** and **G** - Confocal immunocytochemistry indicate cytosolic and limited nuclear localization of AK1 (green) in ES cells (nuclear staining with DAPI, blue) and AK1 association with the cell membrane (G). **H** – CM have greater AK1 levels concentrated in the nucleus, perinuclear space and along myofibrils. **I** – In ES cell-derived CM, AK1 is present in intercellular nanotubular connections and nucleus (inset). **J** and **K** - AK2 is found in the cytoplasm both in ES cells and CM; higher levels of AK2 in CM formed a punctate pattern within the cytoplasm consistent with mitochondrial localization. **L** and **M** - The cytoplasmic localization of the AK5 isoform did vary between ES cells and ES-derived CM; AK5 protein was low in CM. **F** to **M** – representative images of n = 5–10. All scale bars are 10 µm. * *P*<0.05.

Transcriptional profiling revealed a dynamic regulation of adenylate kinase genes associated with differentiation of embryonic stem cells into cardiomyocytes (Fig. 1A). Compared to the pluripotent stem cell source, cytosolic AK1 and AK5 isoforms were upregulated, while mitochondrial AK2 and AK4 and cell motility-associated AK7 were downregulated in cardiomyocytes (Figure 1A). Total adenylate kinase activity was doubled, from 0.1±0.01 µmol/min/mg protein in embryonic stem cells to 0.2±0.004 µmol/min/mg protein in cardiomyocyte progeny (n = 3 per group; Figure 1B). Increased expression was ascribed to marked upregulation of the AK1 isoform as relative *AK1* transcript levels doubled with cardiac differentiation, from 1.04±0.05 units in stem cells to 2.23±0.1 units in cardiomyocytes (n = 3 per group; Fig. 1C left), corroborated by significant increase of AK1 at protein level (Fig. 1C middle). The integrated density of AK1 bands, normalized to α-tubulin levels, was 0.07 for stem cells and 0.40 for cardiomyocytes (Fig. 1C right). Although the *AK2* transcript number was lower in cardiomyocytes (0.63) than stem cells (1.04), AK2 protein levels were stable during differentiation (1.11 vs. 1.25; Fig. 1D). There were more *AK5* transcripts in cardiomyocytes (1.1±0.47 vs. 4.08±0.18), but protein levels of AK5 moderately decreased, from 1.26 in stem cells to 0.93 in cardiomyocytes, relative to α-tubulin (Fig. 1E). The difference in adenylate kinase isoform transcripts and protein levels indicates possible involvement of miRNAs in patterning adenylate kinase isoforms, and regulating cell differentiation [41].

In embryonic stem cells, AK1 was localized in the cytoplasm associated with cell membrane, with limited presence in the nuclear compartment where it appeared excluded from the nucleolus (Fig. 1F and 1G). In stem cell-derived cardiomyocytes, abundant AK1 was concentrated in the perinuclear space, and arranged along myofibrils and sarcolemma (Fig. 1H). Membrane localization of AK1 could be attributed to the AK1β isoform which supplies energy and delivers metabolic signals to membrane metabolic sensors [42], [43]. Expression of membrane-bound AK1β is linked to p53-dependent regulation of cell cycle and cell growth [44]. A greater nuclear presence of AK1 was also characteristic for stem cell-derived cardiomyocytes (Fig. 1I inset). Furthermore, AK1 was concentrated within intercellular nanotubular connections (Fig. 1I) which contribute to cardiomyocyte differentiation [45]. The majority of AK2 was cytoplasmic, both in stem cells and cardiomyocytes (Fig. 1J and 1K), with punctate patterning consistent with mitochondrial localization (Fig. 1K). Such distribution of adenylate kinase isoforms is coherent with formation of a continuous phosphotransfer network, mediating energy transfer and metabolic signaling between cell compartments [7], [46], [47]. The cytoplasmic localization of the AK5 isoform did not vary between stem cells and derived cardiomyocytes (Fig. 1L and 1M). Consistent with Western blots (Fig. 1E), immunostaining for AK5 was low in cardiomyocytes (Fig. 1M). These data are in line with increased adenylate kinase activity demonstrated in postnatal heart development [48] and cell differentiation, notably through an AK1 upregulation [49]. Thus to meet energy and metabolic signaling requirements of developing cardiomyocytes, cardiac differentiation of stem cells is associated with a restructured adenylate kinase transcriptome and proteome.

### Developmental enhancement and redistribution of AK1 in cardiogenic differentiation

**Figure 2:**
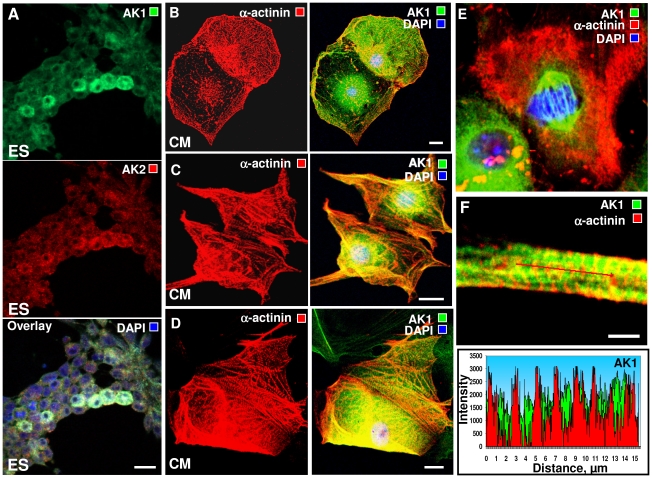
Developmental enhancement and changes in intracellular distribution of adenylate kinase during cardiogenic differentiation and maturation. **A** – Confocal immunocytochemistry of AK1 (green) and AK2 (red) enrichment among ES cells at days 1–2 of differentiation (nuclear staining with DAPI, blue; in the presence of 2.5 ng/mL TGF-β). **B** – Asymmetrical differentiation and maturation of daughter CM correlate with development of a myofibrillar mesh (α-actinin staining, red) and distribution of AK1 (AK1 staining, green) from the perinuclear space to the myofibrillar compartment and throughout the cell. **C** – CM that mature into functional cardiomyocytes have similar myofibrillar (red) and AK1 (green) distribution. **D** - Maturation of cardiomyocytes is supported by development of a myofibrillar network (α-actinin staining, red), intercalation of AK1 (green) with myofibrils, and cytosolic distribution. **E** - During metaphase, AK1 (green) redistributed into cell nucleus and associated with centromeres in contrast to low nuclear AK1 levels in non-dividing cells (red - α-actinin staining). **F** - High abundance of AK1 (AK1 - green, α-actinin- red, upper panel) and tight intercalation with myofibrils (AK1 - green, α-actinin - red, lower panel) in mature ES cell-derived CM. Representative images of n = 6–9. All scale bars are 20 µm, except in E, which is 5 µm.

Early stages of differentiation (day 1–2) were marked by the appearance of AK1 enriched cells, underscoring initial metabolic rearrangement (Fig. 2A). Progression of cardiac differentiation correlated with development of a myofibrillar mesh (α-actinin staining) and redistribution of AK1 from the perinuclear space (Fig. 2B lower cell) to the myofibrillar compartment and the entire cell volume (Fig. 2B upper cell). AK1 distributed asymmetrically between daughter cells according to the degree of differentiation (Fig. 2B). Asymmetric differentiation is a proposed mechanism for generation of cell types by unequal inheritance of cell fate determinants [50]. When daughter cells progressed with synchrony into functional cardiomyocytes, AK1 distributed equally (Fig. 2C). Maturation of cardiomyocytes was supported by development of the myofibrillar network with intercalation of AK1 within myofibrils and distribution through cell compartments (Fig. 2C and 2D). Capturing cardiomyocytes in mitosis, revealed AK1 distributed in the nucleus where it associated with centrosomes and the mitotic spindle in metaphase (Fig. 2E). Nuclear translocation of AK1 in metaphase would provide energy support for chromosome disjunction and powering of cell division, in line with the role for adenylate kinase in motility of cilia and flagella which have a 9+2 microtubular structure similar to that of mitotic spindles [51]. In mitotic spindles, adenylate kinase is in close vicinity of motor proteins, as in flagella, associating with the dynein complex to provide “on site” ATP fueling [23], [51]. Adenylate kinase also supports nucleocytoplasmic exchange of macromolecules and regulates nucleotide ratios necessary for DNA and RNA synthesis [16], [52]. In non-dividing cells, a limited presence of AK1 was noted in the nucleus (Fig. 2E left corner), as well as in nuclei of mature adult and mitotically silent cardiomyocytes (not shown). AK1 abundance (Fig. 2F, green, upper) and tight intercalation within the energy-consuming contractile apparatus (Fig. 2F, red, lower) underscores a growing energetic role in active cardiomyocytes. Developmental enhancement of AK1 and deployment in myofibrillar, sarcolemmal and perinuclear/nuclear compartments, as well as targeted redistribution associated with cell cycle, highlights the dynamics of energetic/metabolic signaling circuits in stem cell cardiac differentiation.

### Engagement of AMP/AMPK signaling in stem cell cardiac differentiation

**Figure 3:**
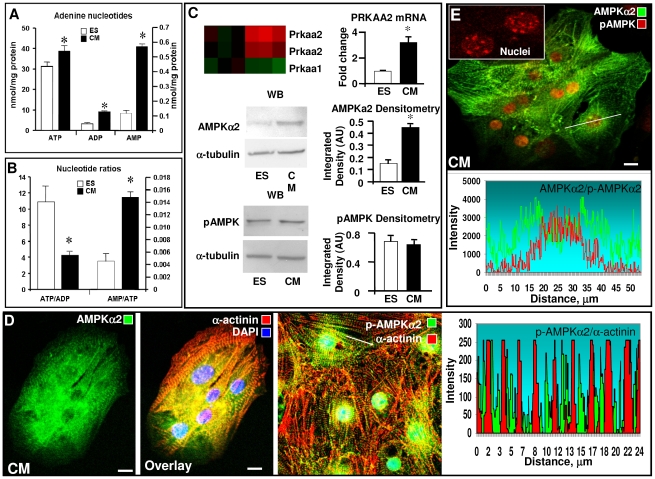
Developmental enhancement of AMP signaling and intracellular distribution of AMPKα2 and the activated form p-AMPKα(Thr172) during stem cell cardiac differentiation. **A** - Compared to ES cells, derived CM have significantly higher total cellular ATP, ADP and free AMP levels. **B** - The ATP/ADP ratio was lower while the AMP/ATP ratio was markedly increased in CM compared to ES cells. **C** - mRNA levels of *Prkaa2*, the gene encoding the major cardiac AMPKα2 catalytic subunit, increased >3-fold upon cardiac differentiation while mRNA levels for *Prkaa1,* encoding the α1 catalytic subunit of AMPK, were down regulated. Protein level of AMPKα2, determined by Western blots (WB) and normalized to α-tubulin amount, was more abundant in CM. The amount of phosphorylated p-AMPKα(Thr172) was not significantly different between ES and CM. **D** - Immunocytochemical confocal microscopy images of AMPKα2 (green) distribution in ES-derived CM and intercalation with myofibrils (α-actinin, red). **E** – Co-immunostaining of CM for AMPKα2 and p-AMPKα(Thr172) indicated significant compartmentalization of AMP signaling with p-AMPKα(Thr172) localized predominantly in nuclei and concentrated in specific nuclear zones (inset). Representative images of n = 5–7. All scale bars are 10 µm.

**Figure 4:**
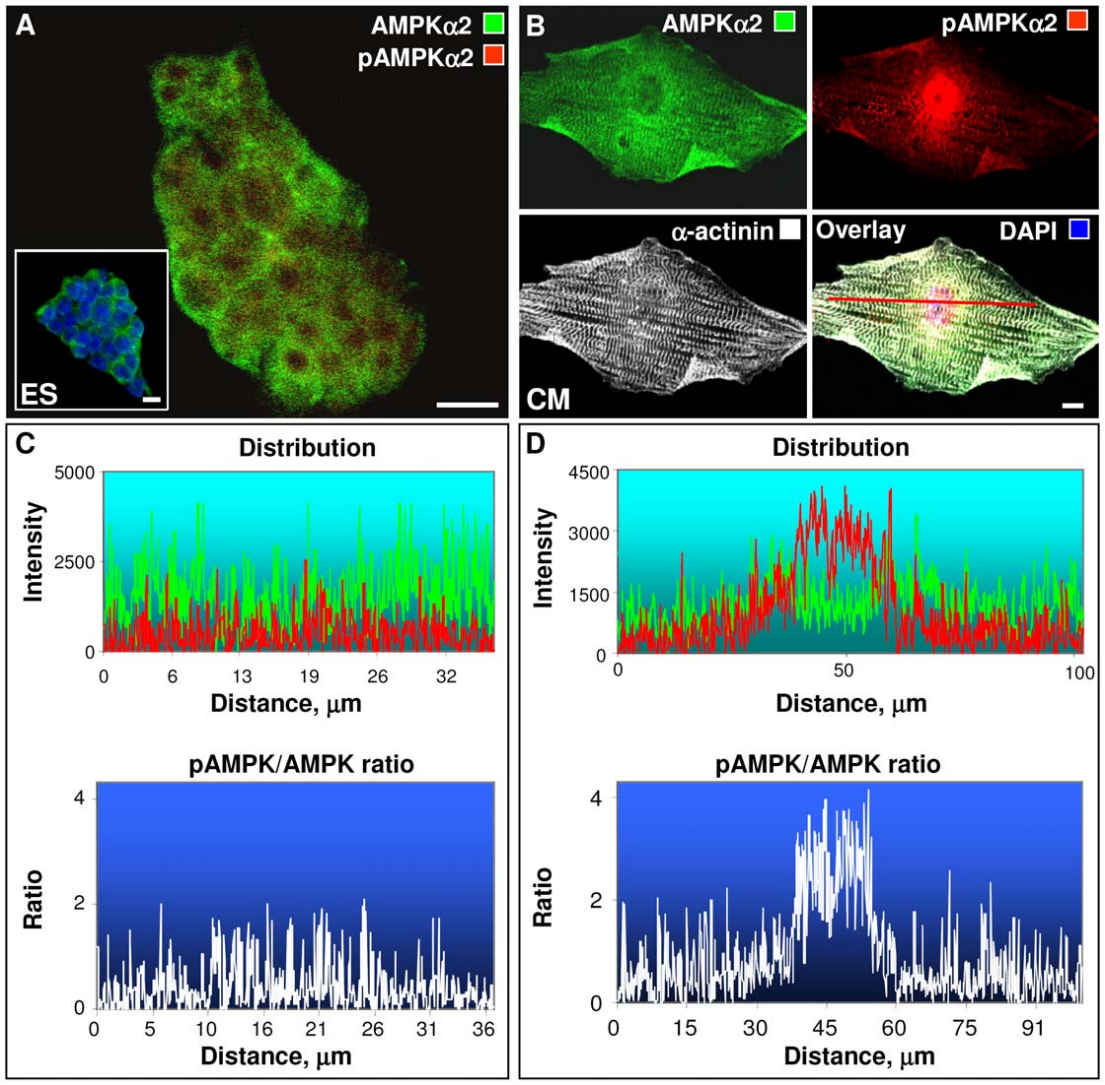
Confocal distribution scans of AMPKα2 and p-AMPKα(Thr172) in stem cells and derived cardiomyocytes. **A** - Confocal images of AMPKα2 (green) and p-AMPKα(Thr172) (red) in ES cells revealed a restricted punctate cytosolic pattern with little presence in nuclei and nucleolar exclusion; p-AMPKα(Thr172) could not be detected in nucleus. **B** - ES-derived CM have higher AMPKα2 (green) and p-AMPKα(Thr172) (red) abundance with characteristic myofibrillar (α-actinin, white) alignment. The p-AMPKα(Thr172) presence in the nucleus was augmented. **C** - Confocal distribution scans of ES cells indicate uniform arrangement of AMPKα2 (green) and p-AMPKα(Thr172) (red) through cell clusters (upper panel) with low p-AMPK/AMPK ratio (lower panel). **D** - Confocal distribution scans of ES-derived CM indicate increase in phosphorylated p-AMPKα(Thr172) (red) in nuclear and perinuclear spaces (upper panel). The p-AMPK/AMPK ratio was higher in the CM nucleus compared to cytosol (lower panel). Representative images and scans of n = 4–6. All scale bars are 10 µm.

Concomitant to deployment of the AMP generating adenylate kinase isoform network, cardiac stem cell differentiation was associated with augmented AMP nucleotide signaling, increased expression of the AMP-sensor AMPKα2, a major cardiac isoform, and nuclear redistribution of the activated and signaling competent p-AMPKα(Thr172). Cardiac differentiation induced significantly higher ATP levels, which increased from 29.6±2.5 nmol/mg protein in stem cells to 38.8±2.6 nmol/mg protein in derived cardiomyocytes (p<0.03, n = 4–11; Fig. 3A). Total ADP levels nearly tripled, from 3.2±0.3 to 9.2±0.4 nmol/mg protein in stem cells and cardiomyocytes, respectively (p<0.01, n = 4–11). Correspondingly, the ATP:ADP ratio was halved in cardiomyocytes (4.2±0.1) compared to stem cells (10.9±1.9, p = 0.01, n = 4–11; Fig. 3B), indicating a higher turnover of adenine nucleotides induced by cardiac differentiation. Cell-free AMP levels, calculated from the adenylate kinase equilibrium, were significantly increased in cardiomyocytes (0.57±0.02 nmol/mg protein) compared to parental stem cells (0.12±0.02 nmol/mg protein; Fig. 3A). The corresponding AMP:ATP ratio, governing the activity of metabolic sensors such as AMPK, were markedly elevated in derived cardiomyocytes (0.015±0.001) compared to the stem cell source (0.005±0.001, p<0.01, n = 4–11). In fact, differentiation of stem cells into cardiomyocytes was associated with distinct transcriptional regulation of AMPK genes (Fig. 3C). The mRNA levels of *Prkaa2*, which encodes the major cardiac AMPKα2 catalytic subunit, tripled from 0.98±0.05 units in stem cells to 3.21±0.44 units in derived cardiomyocytes. Conversely, mRNA levels for *Prkaa1,* encoding the α1 catalytic subunit of AMPK, were downregulated with cardiac differentiation (Fig. 3C). Protein levels of AMPKα2, determined by Western blot, were also more abundant in cardiomyocytes (Fig. 3C middle). The integrated density of AMPKα2 bands, normalized to α-tubulin levels, was on average 0.15 for stem cells, and increased to 0.44 for cardiomyocytes (Fig. 3C middle right). Association and intercalation of AMPKα2 with myofibrils was evident in α-actinin co-stained cardiomyocytes (Fig. 3D right). Although total phosphorylated p-AMPKα(Thr172) was not significantly different between stem cells and cardiomyocytes (Fig. 3C lower), immunocytochemical confocal microscopy revealed a significantly larger nuclear presence of p-AMPKα(Thr172) in derived cardiomyocytes (Fig. 3D and E). Co-staining of cardiomyocytes for p-AMPKα(Thr172) and AMPKα2 indicated significant nuclear co-localization of AMP signaling with p-AMPKα(Thr172) (Fig. 3E). Within the nucleus, p-AMPKα(Thr172) concentrated in intra-nuclear zones to form a speckle-like pattern (Fig. 3E insert), associated with functional activity in the nucleus [53]. In stem cells, AMPKα2 staining was limited to a punctate cytosolic pattern with little presence in nuclei (Fig. 4A), and notably p-AMPKα(Thr172) was absent from stem cell nuclei (Fig. 4A; DAPI co-stain in inset). In contrast, cardiomyocytes displayed higher AMPKα2 and p-AMPKα(Thr172) abundance with augmented p-AMPKα(Thr172) in the nuclear compartment (Fig. 4B). In the cytosol, both AMPKα2 and p-AMPKα(Thr172) showed higher presence in the perinuclear space, and alignment with myofibrils visualized by α-actinin staining (Fig. 4B). Myofibrillar positioning of AMPK would facilitate activation of glycolysis/glycogenolysis for energy supply to contracting sarcomeres, and promote phosphorylation and mechanical performance of contractile proteins [18], [54], [55]. Further delineating AMP-signaling topography, confocal scans revealed uniform arrangement of AMPKα2 (green) and p-AMPKα(Thr172) (red) through stem cell clusters (Fig. 4C). The ratio of p-AMPK/AMPK, an indicator of AMPK activity, was low in stem cells in the 0.10–0.15 range, and evenly distributed (Fig. 4C lower) indicating quiescence of the AMP-signaling system. Conversely, scans of derived cardiomyocytes showed increased p-AMPKα(Thr172) (red) in the nuclear and perinuclear space (Fig. 4D), implicating a localized activation of the AMPK signaling cascade. Notably, total AMPKα2 levels (Fig. 4D green) were not higher in nuclear compared to perinuclear space, suggesting that p-AMPKα(Thr172) may have preferential access to nuclear import mechanisms. The ratio of p-AMPK/AMPK in the cardiomyocyte cytosol was higher at 0.3-0.5, and was further elevated to 2.5–3.5 in the nuclear compartment (Fig. 4D lower). Thus, cardiac stem cell differentiation is characterized by marked activation of the AMP-AMPK signaling cascade manifested by increased AMP/ATP ratio, expression of AMPKα2, and redistribution of p-AMPKα(Thr172) into the nucleus. Activation of the AK-AMP-AMPK signaling axis exerts effects on glucose and lipid metabolism, gene expression, protein synthesis and mitochondria biogenesis [2], [55], [56], [57]. Nuclear translocation of AMPK could thus mediate gene expression by regulating phosphorylation and acetylation of nuclear transport and transcription factors involved in cardiac differentiation, such as the central developmental regulator Mef2C [54], [58], [59], [60]. In this regard, AMPK regulates protein acetylation through altered NAD^+^ levels and NAD^+^-dependent type III deacetylase SIRT1 [61], and through phosphorylation of class IIa histone deacetylase HDAC which adjusts Mef2 transcriptional activity [62]. The present data supports the notion that localized and regulated AMP/AMPK signaling contributes to stem cell cardiac differentiation.

### Adenylate kinase knockdown disrupts stem cell cardiac differentiation

**Figure 5:**
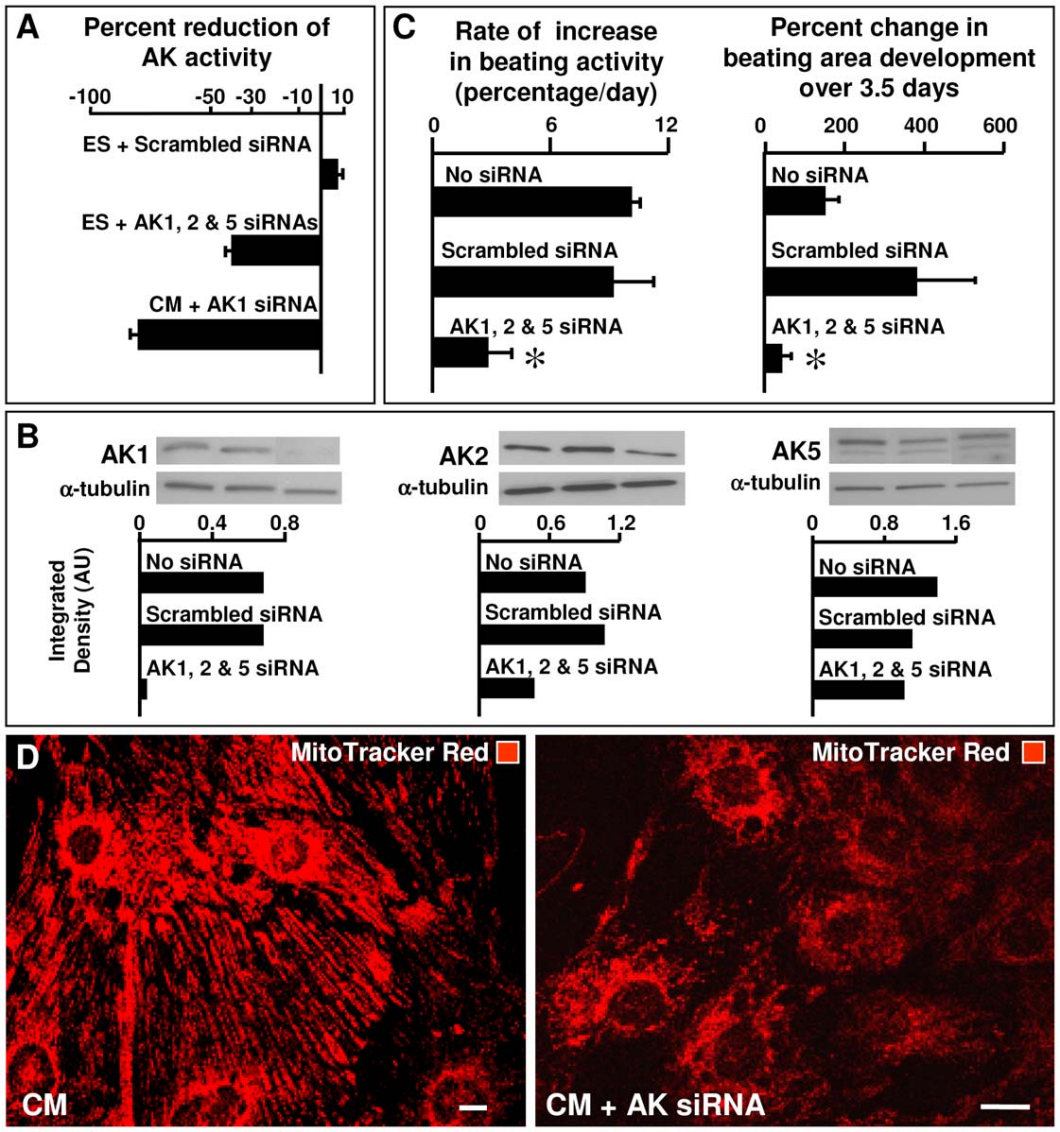
Beating activity and mitochondrial network formation are disturbed by siRNA knockdown of adenylate kinase isoforms. **A**– Adenylate kinase activity was reduced when siRNAs targeting AK1, AK2 and AK5 isoforms were transfected into ES cells and ES-derived CM. **B** - Western blots after combinatorial adenylate kinase siRNA knockdown in ES cells. **C** - Untransfected ES cells and those transfected with a scrambled sequence siRNA were equally efficient at embryoid body generation. Yet, development of beating areas (right) and increase in activity (left) was stunted by simultaneous knockdown of AK1/AK2/AK5 isoforms. **D** - Control ES-derived CM developed organized mitochondrial network but CM derived from AK1/AK2/AK5 siRNA knockdown ES cells had immature and poorly organized mitochondrial networks. Representative data and images of n = 3–5. All scale bars are 10 µm. Star represents statistical significance at *P*<0.05.

Since transcripts of AK1 and AK5 increase with cardiogenesis and AK2 is the predominant mitochondrial isoform, the AMP-generating adenylate kinase system was targeted using a combination of AK1-AK2-AK5 siRNAs. Transfection of embryonic stem cells with AK1-AK2-AK5 siRNAs reduced adenylate kinase activity on average by 40% (n = 3) compared to no siRNA or scrambled siRNA treatment (Fig. 5A). Transfection of cardiomyocytes, isolated from embryoid bodies, with AK1 siRNA reduced adenylate kinase activity by 79% (Fig. 5A), indicating the feasibility to manipulate adenylate kinase isoform levels with siRNAs in stem cells and derived cardiomyocytes. Western blots confirmed that protein levels of AK1, AK2 and AK5 isoforms were diminished on average by 95%, 48% and 27%, respectively, in stem cells 48 h following siRNA transfection (Fig. 5B). Untransfected stem cells gave rise to embryoid bodies that displayed an average daily rate of beating activity increase of 10.1±0.4% (Fig. 5C). Similarly, cells transfected with scrambled siRNA gave rise to embryoid bodies that had an average daily rate of increase in beating activity of 9.2±2% (Fig. 5C). In contrast, AK1-AK2-AK5 siRNA transfected embryoid bodies had a significantly lower average daily rate of increase in beating activity, down to 3±1% (Fig. 5C). By comparing the change in beating activity from the first detection of beating activity to day 12, the percent change in beating area formation remained high in embryoid bodies derived from untransfected stem cells or those transfected with scrambled siRNA (151.8±33.51 and 382.2±145.3 respectively), but not in those derived from cells transfected with the triple AK1-AK2-AK5 siRNAs (43.9±22.4) (Fig. 5C right). In fact, some embryoid bodies with AK1-AK2-AK5 siRNAs did not initiate beating activity at all; others had significantly delayed beating area formation with contractions detectable only after 9 days of development. Thus, knockdown of three major adenylate kinase isoforms early in stem cell specification compromises subsequent development of functional cardiac beating areas. To gain mechanistic insight into causes of functionally deficient cardiac beating areas, cardiomyocytes were isolated from embryoid bodies after knockdown of AK1, AK2 and AK5 isoforms. Control stem cell-derived cardiomyocytes had an organized and expanded mitochondrial network necessary for energy supply to developing sarcomeres and excitation-contraction machinery (Fig. 5D left). In contrast, mitochondrial network formation was disrupted in cardiomyocytes isolated from AK1/AK2/AK5-knockdown embryoid bodies (Fig. 5D right), and appeared immature with perinuclear mitochondrial clustering and little presence in cytosolic and sarcolemmal compartments (Fig. 5D left). Image analysis indicated that the mitochondrial density was lower, on average by about three fold, in AK1/AK2/AK5-knockdown cardiomyocytes compared to untreated controls. Specifically, the MitoTracker Red fluorescence per imaged cell area decreased from 31.4±2.0 to 10.2±1.2 arbitrary units (n = 5) after siRNA treatment. These data provide direct evidence that adenylate kinase and associated metabolic signaling is essential for cardiac programming, in particular with respect to mitochondrial biogenesis and spatial distribution, both critical in cardiogenesis [9], [63]. Silencing of adenylate kinase isoforms could interfere with AMPK-PGC-1α signaling in mitochondrial biogenesis [57], [63]. Indeed, a lower ratio of p-AMPK/AMPK was detected in the cardiomyocyte nucleus after AK siRNA treatment, which dropped from an average of 3.0 in control to 1.5 in cardiomyocytes isolated from AK1/AK2/AK5-knockdown embryoid bodies. Of note, AK1 knockout mice present no apparent developmental cardiac defects which could be related to overlapping function and compensation provided by complementary adenylate kinase isoforms of which eight are currently known [7], [64]. Deletion of AK2, an isoform silenced here, is embryonically lethal in mice [65] and Drosophila [29] with severe defects in mitochondrial structure and energetics, and human mutations of AK2 are associated with developmental defects [30], [31]. Thus, silencing adenylate kinase isoforms and downstream metabolic signaling interferes with cardiac stem cell programming by disrupting mitochondrial biogenesis and network formation.

### Adenylate kinase siRNA inhibits cardiogenic development

**Figure 6:**
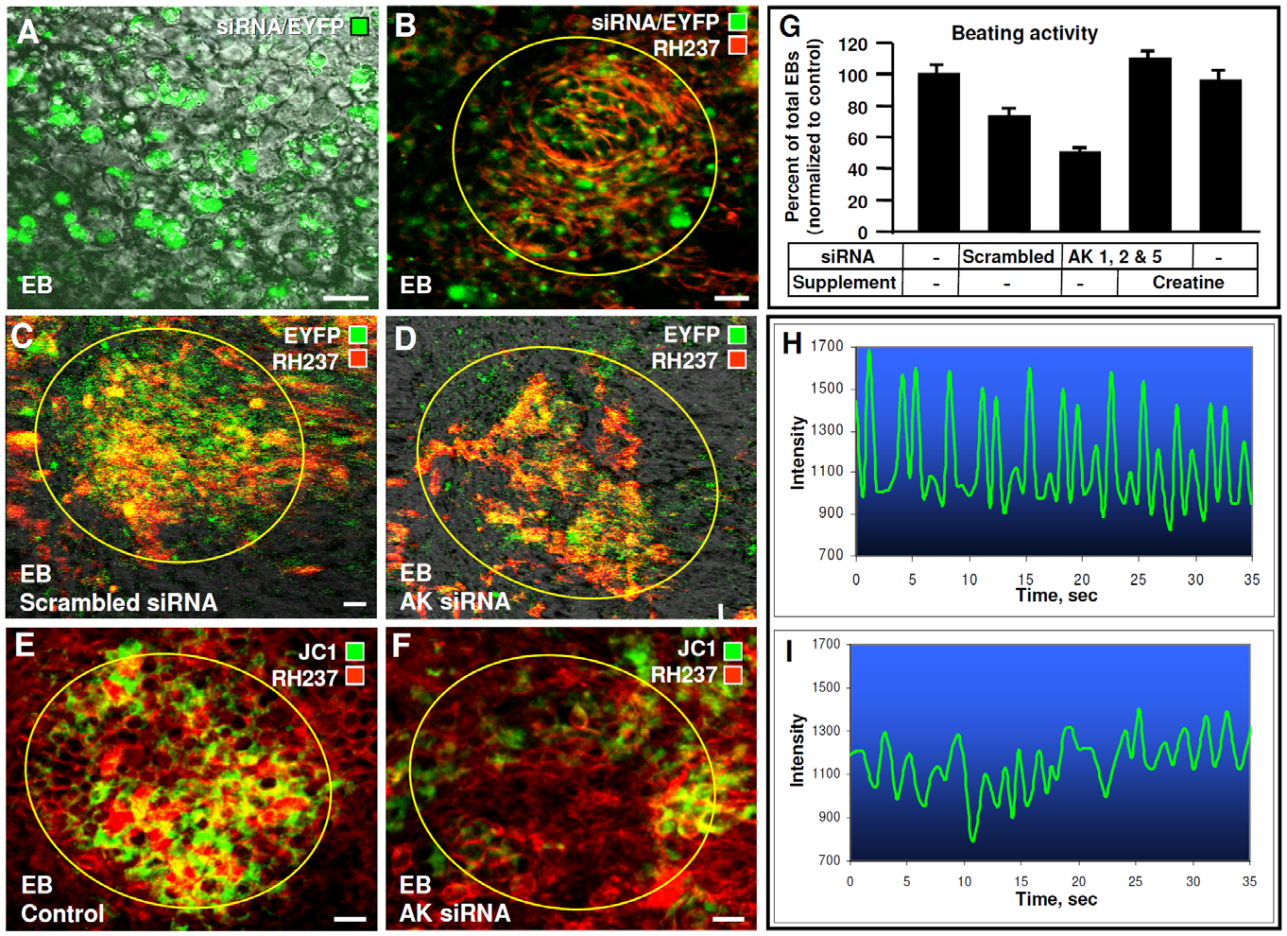
Embryoid bodies transfected with adenylate kinase isoform siRNA cocktail inhibited in cardiac development. **A** – Overlay of transmission (gray) and EYFP (green) images of embryoid bodies transfected with siRNA cocktail and EYFP vector during days 4–6 estimated transfection efficiency at ∼50%. **B** - Transfected cells (green) were found in regions that eventually formed beating areas visualized through increased staining by plasma membrane potential-sensitive RH 237 (red). **C** and **D** – Scrambled siRNA did not affect beating areas (**C**), but the adenylate kinase siRNA cocktail reduced the number and quality of beating areas (**D**). Significant presence of siRNA transfected cells in beating areas visualized by co-transfection with EYFP (green). **E** and **F** - Beating areas visualized using mitochondrial probe JC-1 (green) and RH 237 (red) in control (**E**) and adenylate kinase siRNA cocktail transfected (**F**) embryoid bodies. **G** – Adenylate kinase siRNA cocktail transfection reduced the percentage of beating embryoid bodies compared to no or scrambled siRNA transfections. Creatine supplementation of media, at time of adenylate kinase knockdown, rescued beating. **H** and **I** – Contractions of control beating areas and those affected by adenylate kinase siRNA transfection (lower). All scale bars are 20 µm. Representative images and data of n = 6–8.

Embryoid bodies derived from native stem cells were grown in suspension according to the hanging drop protocol until day 4 when they were allowed to adhere to a gelatin-coated dish. At this point, they were transfected with triple AK1-AK2-AK5 siRNAs and transfection efficiency estimated from co-transfection with an EYFP vector. Approximately 40–60% of the cell population was reverse transfected (green) (Fig. 6A) and found distributed throughout the embryoid body including regions that eventually formed beating areas visualized by increased staining with electrical activity-sensitive RH237 (red) (Fig. 6B). While embryoid bodies transfected with no siRNA or a scrambled sequence had normal beating areas with high electrical activity (red) (Fig. 6C), embryoid bodies transfected with triple siRNA developed sparse and poorly organized beating areas if at all (Fig. 6D). Presence of siRNA transfected cells in beating areas were visualized by co-transfection with EYFP (green) (Fig. 6C and D). As demonstrated previously [9], due to mitochondrial abundance and electrical activity cardiac beating areas are visualized by the mitochondrial probe JC-1 and plasma membrane potential marker RH237 (Fig. 6E and F). Control embryoid bodies had normal beating areas with high mitochondrial density (Fig. 6E), however AK1-AK2-AK5 siRNAs transfected embryoid bodies were unable to form organized cardiac beating areas and displayed low mitochondrial density (Fig. 6F). Quantification of embryoid body beating activity revealed that the percentages of beating embryoid bodies in control, with or without scrambled siRNA, were 74±6 and 100±5% (n = 32), respectively. In contrast, embryoid treatment with AK1-AK2-AK5 siRNAs reduced beating embryoid bodies to 50±7%. Direct measurements of contractile activity, by changes in cell edge position using confocal microscopy, revealed that beating areas with normal adenylate kinase levels had regular contractions with peak amplitudes on average of 700 equivalents, while those with decreased adenylate kinase levels displayed dyssynchronic contractile patterns with peak amplitudes reduced to 400 equivalents. To determine if anomaly in beating area formation was due to a phosphotransfer deficit, culture media were supplemented with creatine to boost a parallel creatine kinase-catalyzed phosphotransfer pathway [16]. By itself, creatine treatment did not significantly affect beating activity as 90±6% of control embryoid bodies (n = 25) remained functionally active (Fig. 6G). However, creatine supplementation to embryoid bodies transfected with AK1-AK2-AK5 siRNAs from the time of adenylate kinase knockdown rescued deficient beating activity, increasing beating percentage to 104±30% (n = 33). Therefore, creatine supplementation, which increases creatine kinase activity and phosphocreatine levels [16], compensated for energetic signaling defects associated with reduction of adenylate kinase isoforms. As creatine increases AMPK activity [66], it is conceivable that the creatine kinase circuit could compensate AK deficit through multiple mechanisms affecting both energetic and metabolic signaling. Although siRNA knockdown of targeted proteins in mammalian cells suppressed gene expression for less than a week (and transfection efficiency was about 50%), this was sufficient to disrupt cardiogenesis and formation of organized beating areas in embryoid bodies. Since embryonic development is a strictly ordered and sequential process of a number of integrated genetic, energetic and signal transduction events, disruption of metabolic signaling during the initial stages could lead to serious consequences in later developmental programming and phenotype maturation [9], [67], [68]. Also, organized cardiogenesis is supported by a cellular network involving interactions of different types of cells, cell movement, intercellular connections and communication by signaling molecules [9], [45]. In this way, disruption of energetic and metabolic signaling in few cells could lead to aberrant cellular network behavior [63]. Thus, intact energetic signaling through the adenylate kinase network is critical in cardiogenesis and formation of the first organized and functional structure in the embryoid body – the cardiac beating area.

### Modulation of cardiogenesis by hyperglycemia and adenylate kinase by cardiogenic TGF-β

**Figure 7:**
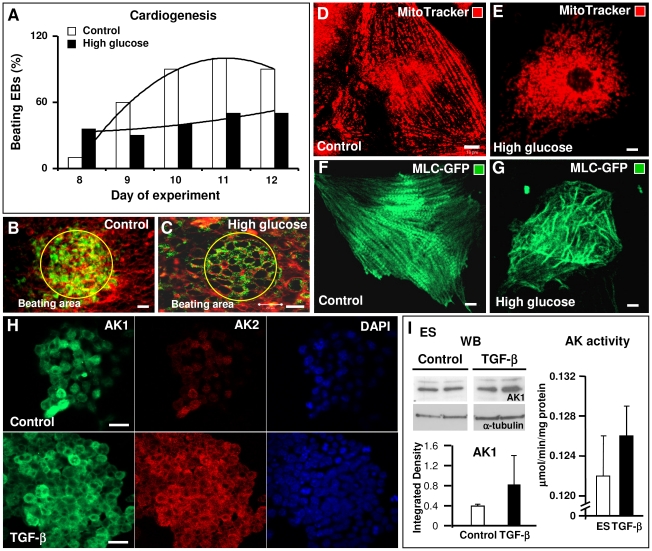
Hyperglycemia disrupts mitochondrial network formation and cardiac myofibrilogenesis, while cardiogenic TGF-β augments adenylate kinase expression. A – Excess glucose (50 mM) stunted beating activity through day 12. **B** and **C** - Beating areas visualized using JC-1 (green) and membrane potential marker RH 237 (red) in control (**B**) and high glucose grown embryoid bodies (**C**). **D** and **E** – Mitochondrial distribution in cardiomyocytes from control (**D**) and glucose-treated embryoid bodies (**E**) visualized using MitoTracker Red. F and G – Myofibrillar formation and organization in control CM (**F**) versus glucose treated CM (**G**) assessed by cardiac specific MLC2v-GFP expression. **H** – Differential AK1 and AK2 immunofluorescent signals in control ES cells (upper) and those treated with TGF-β (lower). **I** – TGF-β treatment increased AK1 protein levels quantified by Western blot (left) and adenylate kinase activity (right). Scale bars in B, C and H are 20 µm, in D, E, F and G - 10 µm. Representative images and data of n = 3–5.

The effects of high glucose include suppression of adenylate kinase phosphotransfer flux and AMPK activity [69], [70], [71]. When embryoid bodies were grown in media with a high concentration of glucose (50 mM), cardiogenesis was impeded as evidenced by blunted beating activity monitored between day 8 to 12 (Fig. 7A). Compared to control, cardiac beating area formation was deficient in high glucose embryoid bodies, which demonstrated lower mitochondrial content (JC1 staining, green) and weaker electrical activity (RH237 staining, red) (Fig. 7B and C). With high concentrations of glucose, the intercalation of the mitochondrial network with myofibrils was lost (Fig. 7D). Mitochondria appeared fragmented (Fig. 7E) while myofibrillar formation and organization, as assessed by MLC2v-GFP expression, became chaotic and considerably more modest (Fig. 7G), in contrast to parallel elongated myofibrils which formed regular patterns (Fig. 7F). Thus, suppression of the AK-AMP-AMPK signaling cascade by siRNA transfection and hyperglycemia produced similar inhibitory effects on mitochondrial network formation and stem cell cardiogenesis. These observations are consistent with reports that hyperglycemic conditions, such as in diabetes, cause mitochondrial fragmentation [72] and lead to embryonic cardiac malformation [73]. Based on previous work, whereby high glucose reduces AMP levels, AK flux and AMPK activity [43], [69], [70], [71], possible mechanisms for the observed effect of hyperglycemia may include diminished adenylate kinase flux and related AMPK-PGC-1α signaling critical in mitochondrial biogenesis [57], [63]. Conversely, to determine whether cardiogenic factors which stimulate stem cell cardiac differentiation [74] modify the adenylate kinase isoform network to achieve their priming effects, embryonic stem cells were treated with TGF-β for two days. Confocal immunocytochemical analysis indicated that AK1 and AK2 signal intensity was stronger following TGF-β treatment (Fig. 7H). AK1 protein levels were higher in stem cells treated with TGF-β (0.825±0.57 AU) versus control (0.389±0.02 AU) when quantified by Western blot (Fig. 7I, left), and total adenylate kinase activity was augmented (0.126±0.003 µmol/min/mg protein) versus control (0.122±0.004) (Fig. 7I, right). These data support the notion that developmental enhancement of adenylate kinase-mediated energetic signaling is an essential event in triggering and promoting stem cell cardiac differentiation and cardiomyocyte maturation.

Our data is in accord with recent studies unveiling the significance and interplay of energetic and metabolic signaling circuits in cellular life. Spatial repositioning of cytosolic adenylate kinase circuit provides energy for cell motility [23] while mitochondrial adenylate kinase AK2 facilitates hematopoietic cell differentiation, unfolded protein response, sound transduction in inner ear and embryonic development [28], [29], [30], [31]. Both AMPK and the upstream kinase LKB1 appear to be critical for embryonic development, maintaining stem cell metabolic homeostasis and supporting cell division [34], [35], [36], [37], [39], [75]. In particular, AMPK was found regulated by the ubiquitin proteasome system and AMPK activation implicated in increased expression of ubiquitin ligases which regulate cardiac transcription factors [76]. Also, it was demonstrated that protein kinase Sik1, a member of the AMPK family, is integral to the genetic network that controls cell cycle, while its absence in embryonic stem cells delays cardiomyogenesis [77]. Cardiac-specific deletion of LKB1, an upstream AMPK kinase, leads to developmental hypertrophy and dysfunction [40], while ablation of another AMPK kinase TGF-β-activated kinase-1, TAK1, causes embryo midgestation lethality [78]. Moreover, signaling through AMPK-family kinases activated by LKB1 regulates myosin phosphatases and cell adhesion [79], further implicating metabolic signaling circuits as common pathways in the etiology of multiple disease states [80].

In summary, this study provides first evidence for the contribution of the adenylate kinase and AK-AMP-AMPK metabolic signaling axis in embryonic stem cell cardiac differentiation. It underscores the significance of nucleocytosolic adenylate kinase-mediated energetic and AMP-AMPK metabolic signaling in guiding asymmetric cell differentiation, mitochondrial biogenesis, and myofibrillar network formation, supporting thereby cell cycle progression during cardiogenesis. Developmental distribution of adenylate kinase and AMPK isoforms fosters formation of a continuous phosphotransfer network, mediating energy transfer and metabolic signaling between cell compartments required for developmental programming. Suppression of the adenylate kinase-dependent AMP signaling cascade and metabolic disturbances, such as hyperglycemia, disrupted maturation of mitochondrial network and myofibrillogenesis, precluding formation and function of organized cardiac beating structures. Collectively, these findings offer a new perspective in the understanding of developmental system bioenergetics and metabolic signaling circuits in cardiac regenerative biology.

## Materials and Methods

### Embryonic stem cell differentiation

Murine embryonic stem cells (CGR8) and those expressing MLC2v-GFP coupled to the cardiac α-actin promoter [81] were maintained in Glasgow Minimum Essential Medium (BioWhittaker-Cambrex) [74], [82]. Cells were differentiated in media containing 20% FBS using a hanging-drop method [9], [83]. Where applicable, media were supplemented with 5 mM creatine, 50 mM glucose or 2.5 ng/mL TGF-β (Sigma-Aldrich). Forming embryoid bodies were grown in differentiation medium, then plated. Beating percentages were determined by light microscopy.

### Cell isolation, enzyme assays and Western blots

Embryonic stem cells and cardiomyocytes isolated by Percoll gradient [9], [83] were extracted in 150 mM NaCl, 5 mM EDTA, 60 mM Tris-HCl (pH 7.5), 0.2% Triton X-100 and protease inhibitors (Complete Mini, Roche). Adenylate kinase activity was measured as described previously [24]. For Western blots, extracts were separated through 12% Tris-HCl SDS-PAGE, transferred to a PVDF membrane and probed with antibodies against AK1, AK2, AK5, α-tubulin, AMPKα2 and pAMPKα (Santa Cruz Biotechnology).

### Metabolic gene profiling

Total RNA isolated from embryonic stem cells or cardiomyocytes was screened using the mouse genome 430 2.0 array (Affymetrix). Expression profiles were analyzed with the bioinformatics software Genespring GX 7.3 (Agilent Technologies). Gene lists were quality filtered to remove genes with expression levels below background, and limited to report genes that changed by 1.5-fold or greater during cardiac differentiation [9], [84], [85], [86].

### Adenine nucleotide measurements

Adenine nucleotide concentrations in cellular perchloric acid extracts were determined by high performance liquid chromatography (Hewlett-Packard Series 1100, Agilent Technologies) using a triethylamine bicarbonate elution buffer (pH 8.8) [24]. Free AMP was calculated from measured ATP, estimated free ADP, and adenylate kinase equilibrium constant according to the equation ([AMP] = (1.05x[ADP]^2^)/[ATP]) [87]. The metabolically active fraction of the ADP concentration was used to indicate the portion accessible to the adenylate kinase reaction.

### Transfection with short interfering RNA (siRNA)

AK1, AK2 and AK5 levels were reduced by isoform specific siRNAs (Ambion Applied Biosystems) transfected using Lipofectamine 2000 (Invitrogen). Briefly, 2 nmol of siRNA were used in 2 mL of growth media with embryonic stem cells at >50% confluence. Knockdown efficiency was assessed by adenylate kinase activity and Western blots 48 h later. In embryoid bodies adenylate kinase levels were reduced by reverse transfections performed on day 4 of the hanging drop method. Simultaneous transfection with pEYFP N1 vector (Clontech) was used to assess transfection efficiency.

### Confocal imaging

For imaging plasma and mitochondrial membrane potentials, cells were incubated for 30 min at 37°C with 1.3 µM RH 237 and/or 0.5 µM JC-1 (Invitrogen). The organization and assembly of beating areas in the developing mesoderm were determined using LSM 510 META Laser Scanning Systems (Carl Zeiss). Contractions in beating areas were measured as change in cell edge position using confocal microscopy, and analyzed with the region of interest function in the software [9]. For mitochondrial network imaging, cells were incubated with 3 µM MitoTracker Red CMH_2_XRos (Invitrogen) for 30 min at room temperature and fixed in 3% paraformaldehyde [88]. Cells were stained with one or more primary antibodies including cardiac α-actinin, AK1, AK2, AK5 (Sigma-Aldrich and Santa Cruz Biotechnology), AMPKα2 and p-AMPKα(Thr172) (Cell Signaling and Abcam), and corresponding secondary antibodies (Santa Cruz Biotechnology).

### Statistics

Comparisons between groups were performed by two-tailed Student's *t*-tests. Data are presented as mean±SEM; *n* refers to sample size. *P*<0.05 was considered significant.
